# Supplementary material for: What Is the Impact of Unemployment as an Adverse Experience? Post-Traumatic Stress Disorder and Complex Post-Traumatic Stress Disorder: A Meta-Analysis
Source: Int J Environ Res Public Health. 2025 Apr 28;22(5):696. doi: 10.3390/ijerph22050696 (PMC12110764; doi:10.3390/ijerph22050696)

Supplementary material

Table S1. Quality assessment tool for observational cohort and cross-sectional studies

Criteria	Yes	No	Other (CD, NA, NR)
1. Was the research question or objective in this paper clearly stated?			
2. Was the study population clearly specified and defined?			
3. Was the participation rate of eligible persons at least 50%?			
4. Were all the subjects selected or recruited from the same or similar populations (including the same time period)? Were inclusion and exclusion criteria for being in the study prespecified and applied uniformly to all participants?			
5. Was a sample size justification, power description, or variance and effect estimates provided?			
6. For the analyses in this paper, were the exposure(s) of interest measured prior to the outcome(s) being measured?			
7. Was the timeframe sufficient so that one could reasonably expect to see an association between exposure and outcome if it existed?			
8. For exposures that can vary in amount or level, did the study examine different levels of the exposure as related to the outcome (e.g., categories of exposure, or exposure measured as continuous variable)?			
9. Were the exposure measures (independent variables) clearly defined, valid, reliable, and implemented consistently across all study participants?			
10. Was the exposure(s) assessed more than once over time?			
11. Were the outcome measures (dependent variables) clearly defined, valid, reliable, and implemented consistently across all study participants?			
12. Were the outcome assessors blinded to the exposure status of participants?			
13. Was loss to follow-up after baseline 20% or less?			
14. Were key potential confounding variables measured and adjusted statistically for their impact on the relationship between exposure(s) and outcome(s)?			

CD=Cannot determine; NA=Not applicable; NR=Not reported.

Table S2. Quality Assessment Tool for Observational Cohort and Cross-Sectional Studies

ID	Authors	Criteria														Score	Quality rating
		Q1	Q2	Q3	Q4	Q5	Q6	Q7	Q8	Q9	Q10	Q11	Q12	Q13	Q14		
01	(Ali et al., 2012)	1	1	1	1	0	0	0	0	1	0	1	0	0	0	6.0	Fair
02	(Astill et al., 2021)	1	1	0	1	0	0	0	0	1	0	1	0	0	1	6.0	Fair
03	(Ayazi et al., 2012)	1	1	1	1	0	0	0	1	1	0	1	0	0	0	7.0	Good
04	(Baek et al., 2022)	1	1	1	1	0	0	0	0	1	0	1	0	0	0	6.0	Fair
05	(Bronner et al., 2010)	1	1	1	1	0	0	1	1	1	0	1	1	0	0	9.0	Good
06	(Cénat & Derivois, 2014)	1	1	1	0	0	0	1	0	0	0	1	0	0	1	6.0	Fair
07	(Cerdá et al., 2013)	1	1	0	1	0	0	0	1	1	0	1	0	0	1	7.0	Good
08	(Cheng et al., 2015)	1	1	0	1	1	0	0	0	1	0	1	0	0	1	7.0	Good
09	Choi et al., 2021	1	1	0	0	0	0	0	0	1	0	1	0	0	1	5.0	Fair
10	(Cohen et al., 2009)	1	1	0	1	0	0	0	1	1	0	1	0	0	1	7.0	Good
11	(Cofini et al., 2015)	1	1	1	1	1	0	0	0	1	0	1	0	0	1	8.0	Good
12	(Eşsizoğlu et al., 2017)	1	1	1	1	0	0	0	0	1	0	1	0	0	1	7.0	Good
13	(Facer-Irwin et al., 2022)	1	1	0	1	1	0	0	0	1	0	1	0	0	1	7.0	Good
14	Frost et al (2019)	1	1	1	0	0	0	0	0	1	0	1	0	0	0	5.0	Fair
15	(Gros et al., 2013)	1	1	1	1	0	0	0	0	1	1	1	0	1	1	9.0	Good
16	(Hyland, et al., 2017c)	1	1	1	0.5	1	0	0	0	1	0	1	0	0	1	7.5	Good
17	(Hyland et al., 2018a)	1	1	1	0.5	0	0	0	0	1	0	1	0	0	1	6.5	Fair
18	(Hyland et al., 2021)	1	1	0	0.5	0	0	0	1	1	0	1	0	0	1	6.5	Good
19	(Karatzias et al., 2019b)	1	1	1	1	0	0	1	0	1	0	1	0	0	1	8.0	Good
20	(Kimerling et al., 2009)	1	1	0	1	0	0	0	0	1	0	1	0	0	0	5.0	Fair
21	(Kvedaraite et al., 2022)	1	1	1	1	0	0	0	0	1	0	1	0	0	1	7.0	Good
22	(Lee et al., 2009)	1	1	0	1	0	0	0	0	1	0	1	0	0	1	7.0	Good
23	(Mills et al., 2016)	1	1	1	1	0	0	0	0	1	0	1	0	0	1	7.0	Good
24	(Murphy et al., 2021)	1	1	1	0.5	0	0	0	1	1	0	1	0	0	1	7.5	Good
25	(Nandi et al., 2004)	1	1	1	0	1	1	0	0	1	0	1	0	1	1	9.0	Good

Note: Ratings were conducted in accordance with the NIH quality assessment tool.

Table S2. Quality Assessment Tool for Observational Cohort and Cross-Sectional Studies

ID	Authors	Criteria														Score	Quality rating
		Q1	Q2	Q3	Q4	Q5	Q6	Q7	Q8	Q9	Q10	Q11	Q12	Q13	Q14		
26	(Pazderka et al., 2022)	1	1	1	0	0	0	0	0	1	0	1	0	0	1	6.0	Fair
27	(Powers et al., 2014)	1	1	1	1	0	0	0	0	1	0	1	0	0	1	7.0	Good
28	(Rybojad et al., 2016)	1	1	1	0	0	0	0	1	1	0	1	0	0	1	7.0	Good
29	(Serrano et al., 2021)	1	1	1	1	0	0	0	0	1	0	1	0	0	1	7.0	Good
30	(Simon et al., 2019)	1	1	1	1	0	0	0	0	1	0	1	1	0	1	8,0	Good
31	(Stuber et al., 2010)	1	1	1	0	0	0	0	0	1	0	1	0	0	1	6.0	Fair
32	(Teramoto et al., 2015)	1	1	0	0.5	1	0	0	0	1	0	1	0	0	1	6.5	Fair
33	Weiss et al (2011)	1	1	1	0	0	0	0	0	1	0	1	0	0	0	5.0	Fair

Note: Ratings were conducted in accordance with the NIH quality assessment tool.

Figure S1. Funnel plot for publication bias between unemployment and the relationship with PTSD.

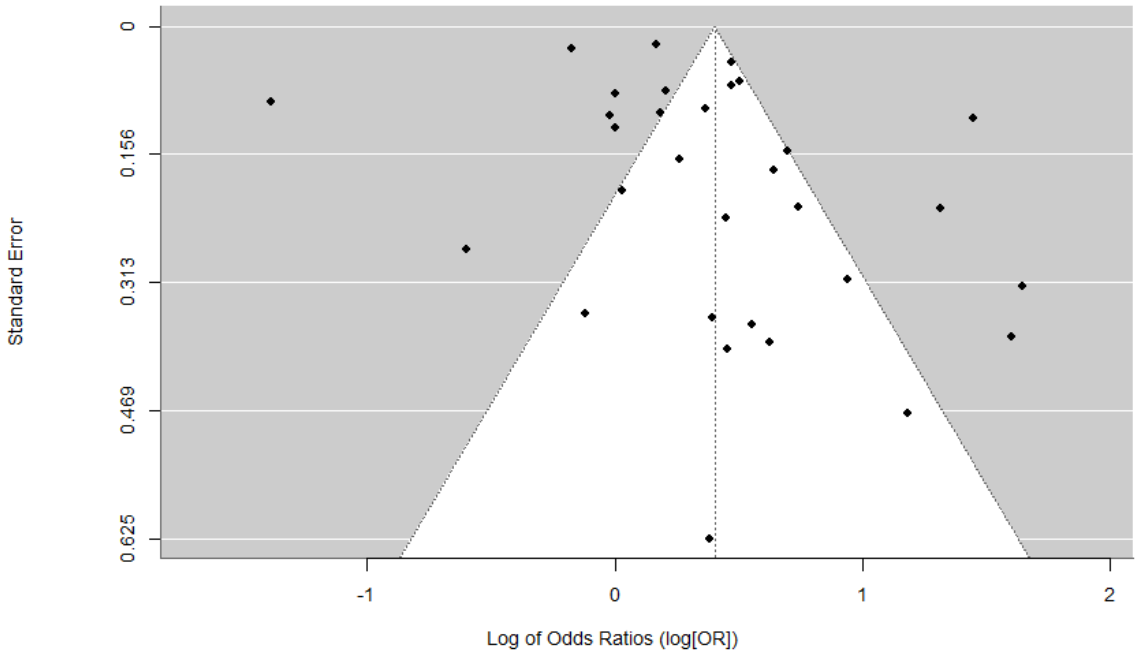

Figure S2. Funnel plot for publication bias between unemployment and the relationship with CPTSD.

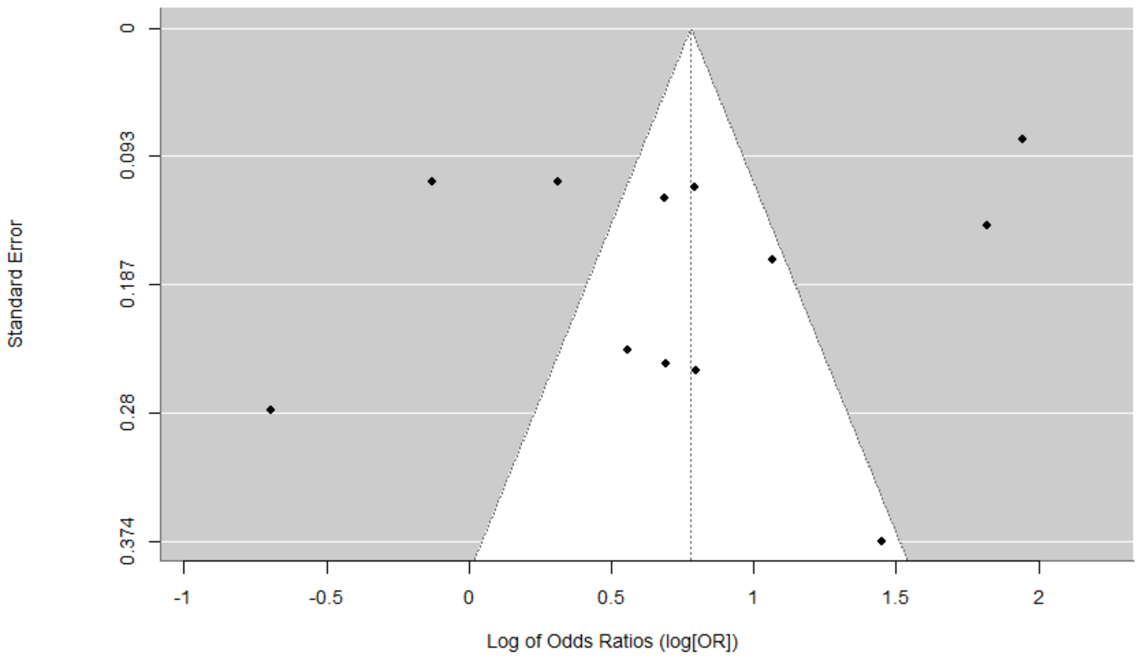

Supplement: Supplementary file 1 [file ijerph-22-00696-s001.zip › ijerph-3516087-supplementary.pdf]
